# Corticospinal mirror neurons

**DOI:** 10.1098/rstb.2013.0174

**Published:** 2014-06-05

**Authors:** A. Kraskov, R. Philipp, S. Waldert, G. Vigneswaran, M. M. Quallo, R. N. Lemon

**Affiliations:** Sobell Department of Motor Neuroscience and Movement Disorders, UCL Institute of Neurology, London WC1N 3BG, UK

**Keywords:** mirror neuron, motor cortex, grasp, hand, corticospinal system

## Abstract

Here, we report the properties of neurons with mirror-like characteristics that were identified as pyramidal tract neurons (PTNs) and recorded in the ventral premotor cortex (area F5) and primary motor cortex (M1) of three macaque monkeys. We analysed the neurons’ discharge while the monkeys performed active grasp of either food or an object, and also while they observed an experimenter carrying out a similar range of grasps. A considerable proportion of tested PTNs showed clear mirror-like properties (52% F5 and 58% M1). Some PTNs exhibited ‘classical’ mirror neuron properties, increasing activity for both execution and observation, while others decreased their discharge during observation (‘suppression mirror-neurons’). These experiments not only demonstrate the existence of PTNs as mirror neurons in M1, but also reveal some interesting differences between M1 and F5 mirror PTNs. Although observation-related changes in the discharge of PTNs must reach the spinal cord and will include some direct projections to motoneurons supplying grasping muscles, there was no EMG activity in these muscles during action observation. We suggest that the mirror neuron system is involved in the withholding of unwanted movement during action observation. Mirror neurons are differentially recruited in the behaviour that switches rapidly between making your own movements and observing those of others.

## 1. Introduction

Since their discovery in the early 1990s, mirror neurons have been at the centre of neuroscientific debate, reflecting the wide variety of functional roles proposed for them. One of the proposed functions relates to the role of mirror neurons in understanding the goal of an observed motor act [1]. There are two key points here. Mirror neuron discharge begins at short latency after commencement of the observed action, suggesting a rather low-level system, not dissimilar to that of premotor canonical neurons that respond at short latency to vision of graspable objects. The second point is that complex movements, such as grasp of an object, with its inherent high degrees of freedom, may be quite difficult to ‘understand’ or ‘classify’ in purely sensory terms (e.g. from visual information about the position and movements of the digits, for example) but can be readily defined with the involvement of one's own motor system and its constituent mirror neurons.

It is a general rule in neuroscience that the role of a particular brain area or type of neuron must be defined in terms of functional connectivity, which both illuminate and constrain theories about possible function. The discovery of mirror neurons in area F5 of the ventral premotor cortex, a key node in the ‘visuomotor grasping circuit’ [2], fitted with the involvement of the motor cortex in action observation. However, mirror-like activity has now been discovered in primary motor cortex (M1; [3,4]) and in other cortical and sub-cortical areas [5–10], prompting questions as to whether similar or different functions are served by mirror neurons in these structures.

Even in area F5, the identity of mirror neurons has not been clarified: are they pyramidal neurons or interneurons, in which layer(s) are they found, and can their properties and function be explained in terms of the connections they make? In this paper, we report the existence of identified corticospinal neurons in F5 with mirror-like properties. As area F5 gives rise to only a small proportion of the corticospinal projection, an obvious question was whether M1 corticospinal neurons also showed evidence of mirror-like activity. The answer is clearly yes, and so we also report the properties of these M1 mirror neurons. We also make a preliminary attempt at comparing and contrasting mirror neuron pyramidal tract neurons (PTNs) in these two cortical areas.

The discovery that PTNs have mirror neuron properties raises at least two important issues. First, it demonstrates that even the executive components of the motor cortex are involved in situations in which we observe the actions of others, and second, it emphasizes that the absence of any overt movement during such situations must involve inhibitory mechanisms that allow smooth transitions from execution to observation.

## 2. Experimental procedures

### (a) Monkeys

These experiments involved recordings in three purpose-bred adult macaques (two males, M41 and M47, one female M43).

### (b) Tasks

**Figure 1.**
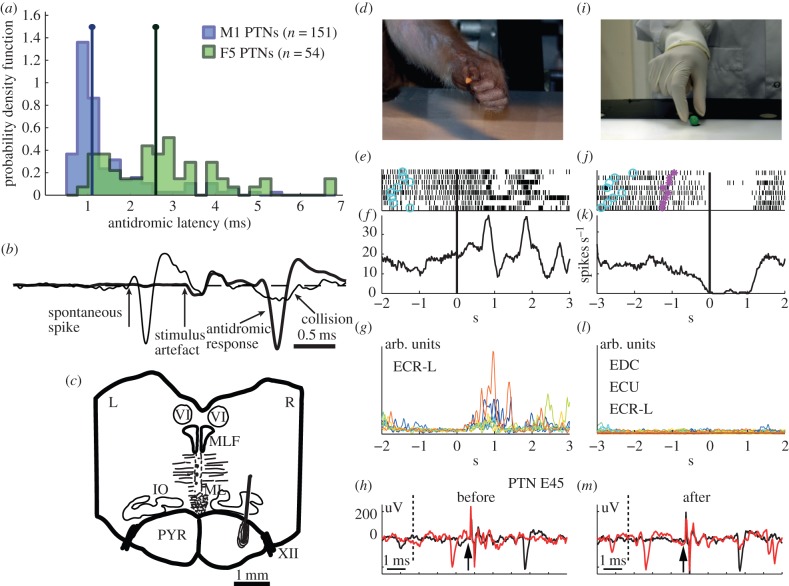
Identification of PTNs with mirror properties. (*a*) Probability density functions comparing antidromic latencies of identified PTNs. Distributions are shown for M1 (blue) and F5 (green) PTNs (bin width 0.25 ms). The two vertical lines correspond to the median antidromic latency for each population of PTNs (1.1 and 2.6 ms for M1 and F5, respectively). The two median values are significantly different (*p* < 0.0001, Wilcoxon rank-sum test) (from Vigneswaran *et al*. [11]). (*b*) Antidromic response of an M1 PTN (heavy trace, average of 40 sweeps). Arrows indicate the onset of the PT stimulus and antidromic spike. The antidromic latency of this PTN was 0.9 ms and spike duration was 0.24 ms. The thin trace shows collision of the antidromic spike with a spontaneous spike from this PTN, which occurred just before the PT stimulus (from Vigneswaran *et al*. [11]). (*c*) Transverse section through brainstem of M41, showing location of tip of posterior PT electrode on right (R) side and surrounding gliosis. PYR, pyramidal tract; IO, inferior olive; ML, medial lemniscus; MLF, medial longitudinal fasciculus; VI, abducens nucleus; XII, hypoglossal nerve. (*d*) Photo of monkey grasping a piece of food in a precision grip; (*e*,*f*) raster plot and averaged firing rate for an F5 PTN during self-grasp aligned to cue for onset of reach-to-grasp movement (indicated by black vertical lines). Note that there were several bursts of activity in the PTN, associated with the initial grasp of the food reward and then release of food at the mouth. Activity of ECR-L (extensor carpi radialis), one of nine simultaneously recorded EMGs is shown in (*g*). (*i*) Photo of experimenter grasping a piece of food in precision grip; (*j*,*k*) raster plot and averaged firing rate for the same F5 PTN during eight trials of observation of precision grip of food, aligned to the moment of contact of the experimenter's hand with the target object (indicated by black vertical lines). Light-blue circles on each trial indicate the beginning of baseline interval for each trial (experimenter's hand motionless in full view of monkey), and magenta asterisks indicate the beginning of experimenter's movement towards the object. In this monkey (M43), a block design was used. (*l*) Superimposed records of EMG activity of all eight observation trials from three extensor muscles (EDC, ECU and ECR-L). Note almost complete absence of EMG activity during action observation. Other six recorded muscles (not shown here) also did not show any significant activity during mirror testing. (*h*,*m*) Antidromic responses from the PTN in response to PT stimulation; the onset of PT stimulus is indicated by arrows, black curves are averages over tens of trials, antidromic spikes had constant latency (2.8 ms) before (*h*) and after (*m*) mirror testing; red curves show collisions when a spontaneous spike appeared after the collision interval (indicated by dotted line): the antidromic spike was collided and absent from the record.

In two monkeys (M41 and M43), the mirror system was investigated using a rather open, clinical testing protocol. For the action execution condition, the monkey grasped a small food reward placed within its peripersonal space on a table in front of it (figure 1*d*). For the action observation condition, the monkey watched a human experimenter carrying out a similar gripping action on food positioned in its extrapersonal space (figure 1*i*; [12]). During these observation trials, the monkey sat quietly with both hands immobile and resting on the table.

The third monkey (M47) was trained on a formal apparatus to test mirror properties, allowing us to compare neuronal activity for grasp of the same object, whether the grasp was made by the monkey or the experimenter [4]. Three different objects were mounted on a carousel device so that they could be presented to either the monkey or the experimenter. The monkey was trained to use a precision grip of a trapezoid-shaped object between index finger and thumb, to displace it in a controlled fashion, hold it steady for 1 s and then release it. Other objects included a sphere, held in a whole-hand grasp, or a ring, held with the index finger in a hook grip. Alternatively, the monkey observed the experimenter perform the same range of grasps applied to the same objects. They were gripped, displaced and held in the same way as the monkey did. Each trial began with the monkey holding down two homepads. In ‘execution trials’, electronic screens were made opaque at trial onset, and the monkey was only able to see which object was to be presented after he had held the homepads down for approximately 0.8 s. After a further variable delay of 0.8–1.5 s, a light-emitting diode (LED) cued the monkey to release one hand to reach, grasp, displace and hold the object. In ‘observation trials’, the monkey had to keep both homepads depressed while the experimenter performed the grasp. A different screen again prevented the monkey seeing which object was to be grasped until approximately 0.8 s after trial onset. Observation trials were aborted if the monkey released either homepad during the experimenter's action. In both versions of the task, monkeys received food rewards after completion of both execution and observation trials. The monkeys were trained to perform the execution version of the task over several months but were not exposed to the action observation protocols until after cortical recordings had begun.

### (c) Cortical recording and antidromic identification of pyramidal tract neurons

Neuronal recording was carried out using Thomas Recording multiple electrode drives targeting either area F5, located in the rostral division of the ventral premotor cortex (M41 and M43), or the M1 hand area (M43 and M47), both contralateral to the grasping hand. All neurons were discriminated using modified Wave_Clus software (see [12] for details). In these experiments, our main objective was to record from PTNs, identified antidromically by stimulation of the pyramidal tract through fine, tungsten electrodes chronically implanted in the pyramidal tract (figure 1*c*) under general anaesthesia. PT electrodes were positioned stereotaxically, and interoperative electrophysiological tests indicated that the electrode tips were in the PT [12]. This was confirmed in subsequent histology for M41 and M43; M47 is still alive.

Figure 1*b* shows an example of an average of 40 antidromic responses in a PTN in M1 (thick trace). The latency of the antidromic response was 0.9 ms. The thin trace shows the results of a collision test confirming the antidromic nature of the response: a spontaneous spike from this neuron, which occurred just before the PT stimulus, collided the antidromic response.

### (d) Eye movements

In M47, we were able to monitor eye movements during both execution and observation trials, using a non-invasive system (ISCAN ETL-200, 120 Hz). There was no requirement in our protocol for the monkey to foveate the equipment. However, the analysis of the monkey's oculomotor behaviour revealed that the monkey spent a significant amount of time with its gaze directed at the object when it first became visible, and at the reach-to-grasp action that followed [13]. While, on average, a greater proportion of time was spent gazing at execution trials (70%) than observation trials (53%) [13], the monkey clearly spent a considerable amount of time attending to the experimenter's object and action, indicating that on most trials the monkey always attended to at least part of the experimenter's action (see [14]). The pattern of gaze behaviour during execution and observation was well correlated (0.92, *p* < 0.05).

### (e) Electromyographic recording

The final important aspect of these studies was to make a rigorous examination of any muscle activity present during action observation. In two of the monkeys in which most of the recordings were made (M43 and M47), we chronically implanted EMG electrodes in multiple arm, hand and digit muscles in the arm used for grasping [12,15]. This allowed us to make simultaneous recordings of all these muscles during action observation and check that the monkeys did not make covert movements with that arm that might explain modulation of PTN discharge.

## 3. Results

### (a) Pyramidal tract neurons in M1 and area F5

In these studies, we recorded from 64 PTNs in F5 and 132 PTNs in M1. In F5, PTNs were recorded at depths of 1–3 mm from the cortical surface. As our penetrations were close to the inferior limb of the arcuate sulcus, this probably indicates that the first PTNs we encountered were located in layer V of the convexity, while those lying deeper were located in the same lamina in the bank of the arcuate sulcus. These PTNs were found at sites at which intracortical microstimulation (ICMS) evoked digit movements with thresholds above 15 µA. No sites yielded mouth or lip movements. Most of the M1 PTNs were recorded from tracks in the anterior bank of the central sulcus and at sites from which digit movements were evoked with ICMS (less than 20 µA, 79%; less than 10 µA, 55%). As can be seen from figure 1*a*, PTNs in these two cortical areas form rather different populations: one in M1 characterized by a large group of fast-conducting PTNs (short antidromic latencies, many less than 1 ms) and the other in F5, with longer latency responses and a broader distribution of antidromic latencies. Mirror neuron properties were found in PTNs with a wide range of antidromic latencies, and so there was no obvious relationship between these properties and conduction velocity. It is important to note that PTNs were selected for further study on the basis of their antidromic response, so the sample was unbiased in terms of the unit's natural activity, which was not tested until stable PTN recording had been achieved. The antidromic response and collision test of each PTN was checked both before (figure 1*h*) and after (figure 1*m*) task performance. This helped in confirming that spikes recorded throughout the execution and observation trials were from the same neuron.

### (b) Absence of hand and digit muscle electromyographic activity during action observation

In the great majority of action observation sessions, the monkey sat calmly throughout and made no hand or arm movements. This was confirmed by inspection of simultaneous EMG recordings in M43 and M47, which showed an almost complete absence of activity in all recorded muscles during the period of action observation (−750 to +750 ms relative to the experimenter's grasp). Figure 1*l* shows all superimposed trials of EMG recordings from some of the sampled muscles; all were essentially flat. In a few sessions in M43, the monkey made some small movements and some EMG was present; PTNs that were recorded in sessions showing such EMG contamination during action observation were excluded from the database. EMG contamination was not found in any of the recordings in M47.

### (c) Mirror neuron pyramidal tract neurons in F5

After the removal of any PTNs recorded during EMG contamination, we were left with 48 PTNs of which 25 (52%) showed statistically significant modulation in their discharge in the 1500 ms period centred on the moment the experimenter first touched the piece of food. Discharge in this period was compared to the baseline level of discharge in the period 750 ms before the experimenter's movement began, when the food reward was present on the table (static presentation period). The experimenter's first contact with the food was signalled by a sensor embedded in the table, which detected the presence of a small magnet located in the finger tip of the experimenter's glove (figure 1*i*). To qualify as mirror neurons, we also had to demonstrate that these PTNs showed significant increases in discharge when the monkey grasped a small food reward with its contralateral hand. Once again, the comparison was relative to the discharge rate during the static presentation period described above.

#### (i) Classical mirror neurons

Of these 25 selected mirror PTNs, 11 showed facilitation of their discharge both during action observation and during the monkey's own grasp. This type of mirror neuron activity we term ‘classical’ in pattern, or F-F type (facilitation during both observation and execution), resembling that first described by Gallese *et al*. [16].

#### (ii) Suppression mirror neurons

A quite different pattern was found for the other 14 PTNs, an example of which is shown in figure 1*d–m*. In this case, the PTN again showed increased bursts of activity as the monkey reached and grasped the food reward (figure 1*d*,*f*). However, during action observation (figure 1*i*), its steady discharge was completely suppressed (figure 1*j*,*k*). This suppression of activity was highly reproducible from trial to trial (figure 1*j*).

### (d) Mirror neuron pyramidal tract neurons in M1

**Figure 2.**
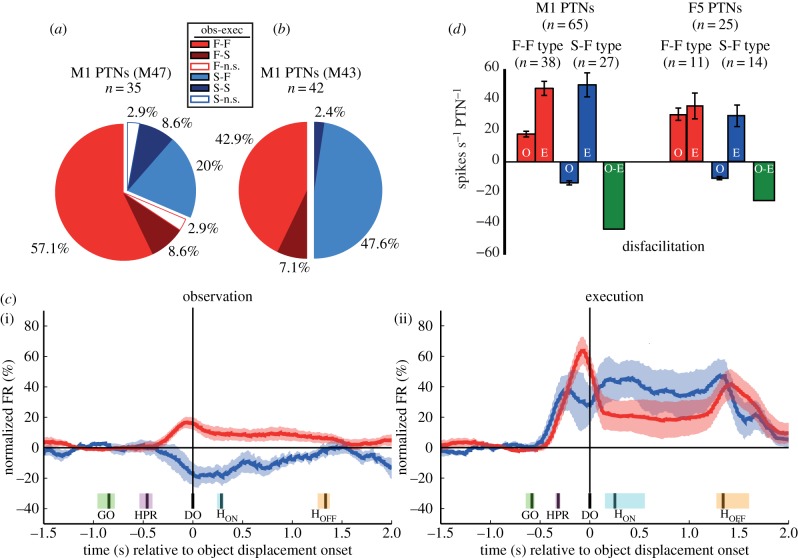
Distribution of different classes of mirror neurons. (*a*,*b*) PTNs modulated during observation. Pie charts showing different types of mirror neurons in M47 (*a*) and M43 (*b*). Some PTNs were facilitated (F-F, light red) during both action observation (O in inset box) and execution (E). Others were suppressed during observation but facilitated during execution (S-F, light blue). Darker shades of both colours indicate a small proportion of PTNs showing suppression during execution and white indicates PTNs with non-significant (n.s.) change in modulation during execution; these PTNs were not included as mirror neurons. (*c*) (i) Population averages during observation for corticospinal mirror neurons (M47) that were activated during execution and whose discharge was significantly facilitated (red, *n* = 20) or suppressed (blue, *n* = 7) during observation (together with s.e.m., shaded areas). Firing rates were normalized to the absolute maximum of the smoothed averaged firing rate of individual neurons defined during execution and observation trials, and the baseline firing rate was subtracted, hence the negative values for the firing rate of suppression mirror neurons. Data aligned to object displacement onset (DO), the median (black line) and the 25th–75th percentile times of other events recorded are shown as shaded areas: go cue, GO (green); homepad release, HPR (magenta); beginning (H_ON_, cyan) and end (H_OFF_, magenta) of the object hold. Firing rates were smoothed using a 400 ms sliding window in 20 ms steps. (ii) Population average for the same groups of mirror neurons during execution. Facilitation-type PTNs showed higher discharge rates during execution compared with observation trials, and suppression-type PTNs changed pattern to facilitation during execution. In this monkey (M47), execution and observation trials were randomized. (*d*) Maximum firing rate of M1 (left) and F5 PTNs (right) during observation and execution trials, expressed as raw firing rates (with s.e.m.). Results from two monkeys (M43 and M47) were pooled for the M1 data; only data from M43 were used for F5. Red bars show average rates for 38 M1 and 11 F5 PTNs facilitated during both observation (O) and execution (E) (F-F type). Note the higher rate of M1 versus F5 PTNs in execution and of M1 PTNs during execution versus observation. Blue bars show rates for 27 M1 and 14 F5 PTNs suppressed during observation (O) and facilitated during execution (E) (S-F type). The green bars show the mean firing rate for these mirror PTNs in observation minus that in execution, to capture the total amount of *relative* disfacilitation in the output from these neurons in M1 and F5 that occurred during observation (partly based on Vigneswaran *et al*. [4]).

After the discovery that PTNs in F5 could show mirror properties [12], it was a natural step to see whether similar responses could be found in M1 PTNs. Of the 132 PTNs recorded, 77 (58%) showed significant modulation during action observation. To reveal this activity in the PTNs recorded in M43 (*n* = 79), we used the same analysis as for F5 (see above). For the other 53 PTNs recorded in M47, we used a one-way ANOVA for three phases of the task: baseline (500 ms before the GO cue), reach (HPR to DO) and hold (H_ON_ to H_OFF_) (see figure 2*c*). We performed a Bonferroni-corrected post hoc test in order to compare the neuronal activity relating to the experimenter's movements (reach, grasp and hold) with the static presentation of the object (baseline).

We were once again able to demonstrate that changes in PTN firing rate during action observation were not associated with movement or low-level muscle activity on the part of the monkey (up to 11 different arm, hand or digit muscles were recorded but were silent during action observation).

The population data for M1 PTNs with mirror-like activity recorded in M47 and M43 are shown in figure 2*a* and *b*, respectively. As in area F5, we also found two main types of mirror neuron activity: those whose discharge was facilitated during both action observation and execution (F-F type, red in figure 2*a*,*b*; 57% (20 PTNs) in M47 and 43% (18 PTNs) in M43); and those whose discharge was suppressed during observation and facilitated during execution (suppression mirror neurons, S-F type, blue; 20% (7 PTNs) in M47 and 48% (20 PTNs) in M43). Thus, of all tested M1 PTNs, 29% (38/132) were facilitation mirror neurons and 20% (27/132) were suppression mirror neurons: a sizeable proportion of PTNs discharging actively during execution exhibited suppression during observation, that is, they reversed their activity.

Note that in a few PTNs discharge was suppressed during execution (F-S type and S-S types, dark red and blue, respectively) or was not significantly modulated during execution (n.s.; two PTNs). We did not count these neurons as mirror neurons.

Figure 2*c* compares the time-resolved normalized firing rates of mirror neurons during observation and execution in M47. We show data from the two main subgroups of PTNs: facilitation mirror neurons that were also facilitated during execution (*n* = 20 F-F type PTNs, red traces in figure 2*c*) and suppression mirror neurons, which reversed their firing pattern and were also facilitated during execution (*n* = 7 S-F PTNs, blue traces). During observation (shown at left), both groups of PTN modulated their background firing rate shortly after the experimenter released the homepad (HPR) to begin their reach-to-grasp action, with peak modulation at the moment when the grasped object was displaced by the experimenter (DO). During execution (shown at right), facilitation PTNs were around three times as active compared with observation; discharge increased to 64% of the maximum modulation above baseline, versus only 17% during observation. The suppression PTNs reversed their pattern of discharge from 19% of the maximum modulation below baseline for observation to 47% above it for execution. Changes in firing rate were sustained at lower levels during the hold period (between event markers H_ON_ and H_OFF_).

### (e) Comparison of mirror pyramidal tract neuron activity in F5 and M1

#### (i) Firing rates: disfacilitation of pyramidal tract neuron output during action observation

In figure 2*d*, we estimate the changes in maximum firing rates (non-normalized) when the task switched from execution to observation. For each PTN, these changes were calculated from the average maximum discharge found during either the monkey's grasp (execution, E) or the experimenter's grasp (observation, O), and expressed relative to the steady rate of discharge recorded during the baseline (static presentation phase), when the monkey first saw the object. We pooled data from the two monkeys (M43 and M47) used for M1 recordings (figure 2*d* left) but we have only used data from M43 for F5 (figure 2*d*, right), because we lacked EMG controls in M41. For M1, we calculated the mean maximum firing rate for 38 F-F-type mirror neurons (red bars), i.e. those facilitated during both observation (O) and execution (E), but much less active in the former condition. The blue bars represent 27 S-F-type PTNs, which were suppressed for observation but facilitated for execution. For all neurons, the baseline firing rate was set at zero, so the discharge rate of suppression mirror neurons has a negative value. The green bar combines results from the two sets of mirror neurons and shows that, *compared with the execution condition*, the population mean firing rate during observation represented a mean disfacilitation of around 45 spikes s^−1^ PTN^−1^. This suggests a major reduction or withdrawal of corticospinal output to the spinal cord during the observation condition.

Figure 2*d*, right, shows a similar calculation for F5 PTNs: 11 F-F type and 14 S-F type. There appear to be two interesting differences with the M1 data. During execution, F5 mirror PTNs discharge at lower absolute rates than M1 PTNs: 35 versus 475 spikes s^−1^ PTN^−1^ in F5 and M1, respectively. The other difference is that, unlike in M1, the firing rates of facilitation-type (F-F, red) mirror neurons were as high during observation as in execution. However, the suppression mirror neurons, by definition, showed little or no activity during observation.

We should stress that this is a preliminary comparison based on data recorded using both the less structured task (M43) and the formal task (M47). A more detailed comparison must await completion of recordings from the second monkey tested with the formal apparatus.

#### (ii) Pyramidal tract neuron activity during different grasp contexts

**Figure 3.**
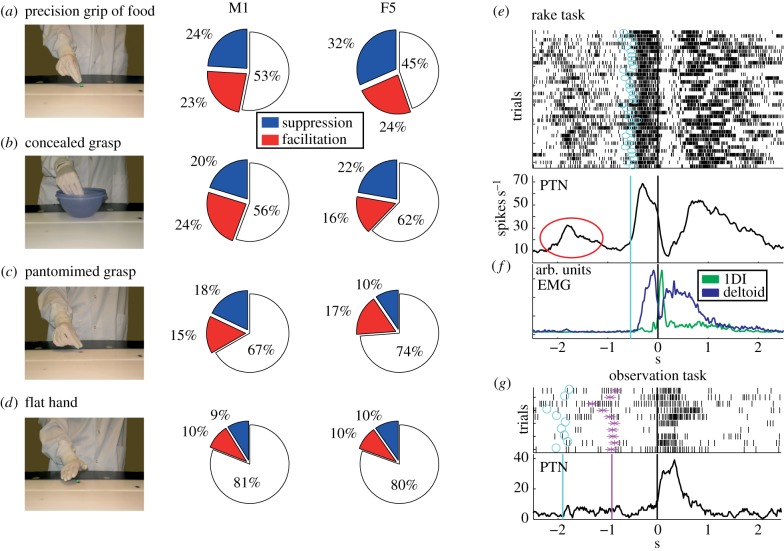
Mirror neuron activity in different behavioural contexts. (*a*–*d*) The proportions of M1 (second column) and F5 (third column) PTNs that showed clear mirror-like activity during observation of grasps carried out in different contexts illustrated in the first column. In all cases, we are reporting percentage of mirror neuron PTNs, which showed significant facilitation (red) or suppression (blue) during the 1500 ms period centred on the experimenter's first contact with the food object (*a*,*b*), or with the table (*c*,*d*), and for which no EMG contamination was present. The numbers of tested PTNs represented in M1 and F5, respectively, were 79 and 38 in (*a*), 79 and 45 in (*b*), 78 and 42 in (*c*) and 79 and 41 in (*d*). (*e*) Activity of an M1 PTN during rake task (Quallo *et al*. [17]) aligned to the moment of the rake crossing the target sensor, blue circles indicate the moment at which the experimenter removed her hand from the homepad. Note the burst of spiking activity (red circle) that occurred during the experimenter's movement and well before any monkey movement. (*f*) EMG activity of distal (1DI) and proximal (deltoid) muscle during the same trials of the rake task. (*g*) Activity of the same PTN during action observation. The spike rasters and average are aligned with the experimenter's contact with the food reward (time 0). Blue circles and magenta asterisks represent the experimenter's homepad presses and releases, respectively.

Figure 3*a*–*d* shows the proportions of F5 and M1 PTNs that showed clear mirror-like activity during observation of grasps carried out in different contexts (illustrated on left) in the experiment carried out in M43, with careful EMG control. These controls resulted in somewhat different numbers of PTNs being available for each test. For precision grip of a small food reward (figure 3*a*), around 47% of M1 and 56% of F5 PTNs showed mirror activity, with similar proportions of ‘facilitation’ (red) and ‘suppression’ (blue) types of mirror neurons in M1, but with more suppression mirror neurons in F5. Discharge was also modulated when the experimenter reached into a small food bowl, concealing the final part of the grasping action from the monkey (figure 3*b*). Many PTNs also modulated their discharge when precision grip was carried out without the food being present (figure 3*c*). In other words, these PTNs also responded when a pantomimed or intransitive action, with no apparent goal, was carried out by the experimenter. The proportion of PTNs responsive to pantomimed or concealed grasp was generally less than that for the precision grip (figure 3*a*–*c*). The smallest number of responsive PTNs was found when the experimenter simply placed her hand flat upon the food reward, in an action that clearly did not involve any kind of grasp (figure 3*d*).

Figure 3*a*–*d* reveals a decrease in the proportion of significantly modulated mirror PTNs responding to the four different tests shown, as the goal of the action became progressively less clear (in the order: precision grip of food, concealed grasp, pantomimed grasp and ‘flat hand’). The decrease was steeper for F5 mirror neurons (falling by 11.7% of PTNs per grasp tested) than for M1 mirror neurons (9.5%). This difference would be worthy of further investigation in the future.

### (f) Rapid switching of mirror neurons between execution and observation states

In these studies, execution and observation trials were kept completely separate. However, two findings showed that mirror neurons can switch rapidly between ‘execution’ and ‘observation’ modes. First, after action observation trials, monkeys M41 and M43 were rewarded 1–2 s after the experimenter's action was complete, and, as expected, mirror neurons showed vigorous discharge as the monkey grasped the food reward (M47 was rewarded by placing the food directly in the monkey's mouth, and generally did not make any digit movements during the reward phase).

Second, in some cases we could detect what appears to be mirror-like activity not long before the monkey executed a movement. The example shown in figure 3*e* is for an M1 PTN recorded in monkey M43 as it used a light rake, held in its left hand, to collect a food reward placed beyond its reach on a table [17]. The rasters and histograms are aligned to the moment when the monkey pulled the rake and food towards itself. The trial began with the experimenter placing the reward on the table, at around –2 s. The PTN showed a brief burst of activity during this period (circled in figure 3*e*), although the monkey was sitting quietly and EMG activity was absent at this time (see records from a digit (1DI) and shoulder muscle (deltoid) in figure 3*f*). The cue for the monkey to move was the experimenter releasing their hand from the food reward (blue circles and line in figure 3*e*): at this point, the monkey picked up the rake, placed the head of the rake beyond the food morsel, pulled the rake plus food back towards itself, released the rake and retrieved the food with its left hand. The monkey's grasp of the rake was associated with bursts of EMG activity in the digit muscle plus a marked increase in the firing rate of the PTN (between −1 and 0 s). The discharge during rake use (up to 65 spikes s^−1^) was higher than that in the early period when the experimenter placed the food on the table (30 spikes s^−1^). Later in this session, we recorded this same PTN while the monkey sat quietly and watched the experimenter grasping. The PTN showed a clear burst of activity during observation of this action (figure 3*g*). Note that the early, circled activity in figure 3*e* is unlikely to reflect a canonical response to the presence of the object, as these responses are mostly lacking in M1 [18] and there was no discernible response of this PTN to the presence of the food object on the table at the beginning of the mirror test shown in figure 3*g*.

### (g) Do cortico-motoneuronal cells show mirror activity?

Some corticospinal neurons terminate directly on alpha motoneurons, and their cortico-motoneuronal (CM) influence can be detected by spike-triggered averaging of EMG [19,20]. We tested the population of PTNs for spike-triggered averaging of EMG. Of the 34 mirror PTNs recorded in M47 tested, five (15%) had clear post-spike effects: three were facilitation and two were suppression mirror neurons. We did not find any clear post-spike effects for PTNs recorded in F5.

## 4. Discussion

Identified PTNs both in area F5 and in the M1 hand area show mirror-like properties. Area F5 was, of course, where mirror neurons were first reported [16,21]. In these early studies, no attempt was made to identify the outputs of these neurons. The discovery that PTNs in area F5 also belong to the mirror neuron population means that activity evoked by action observation is also transmitted to the spinal cord, which therefore could be considered to be part of an extended mirror neuron system.

### (a) Pyramidal tract neurons in F5 as mirror neurons

In area F5, PTNs are quite sparse [22]. PTNs that showed mirror-like behaviour were located close to the inferior limb of the arcuate sulcus and inferior to the arcuate spur (see fig. S1 of [12]). They were recorded at depths of up to 3 mm from the cortical surface. The location fits well with the description of corticospinal neurons, retrogradely labelled from injections in the rostral cervical spinal cord (C3–C5; [23,24]). Importantly, corticospinal neurons are found both in the bank of the arcuate sulcus and on the adjacent convexity of the gyrus [23], where mirror responses have been reported [25].

### (b) Variation in the pattern of mirror neuron activity: the suppression mirror neuron

We discovered PTNs with a new variant of mirror activity, which we termed ‘suppression mirror-neurons’. Unlike the ‘classic’ type of mirror neuron, which shows closely matched increases in discharge during both execution and observation trials (F-F type; see figure 2*d*, right), activity in suppression mirror neurons is either reduced or abolished during action observation (figure 1*j*,*k*). In a strict sense, a mirror neuron should show the same response to both execution and observation. However, we now know that discharge can be significantly altered by changing, for example, the location of the observed action [26], the viewing angle [27] and the reward [28], so this feature of mirror neuron activity is not fixed. Interestingly, the proportions of tested PTN mirror neurons responsive to different grasp contexts is rather similar for both facilitation- and suppression-type of mirror neurons (pie charts in figure 3*a*–*d*).

### (c) Can mirror neuron activity in pyramidal tract neurons result from covert movement?

Because mirror neurons are found within the cortical motor network, there is always the danger that their discharge is not evoked by action observation *per se*, but rather is associated with small movements or adjustments in posture, which the monkey makes while viewing the actions of others. In most published mirror neuron studies, some control EMG recordings have usually been carried out at some time during the study to confirm that this is not the case. However, only EMG data acquired simultaneously with the neural recordings can completely exclude the possibility that covert movements were present. We used this approach for most of our recordings: monkeys M43 and M47 were both implanted with EMG electrodes in digit, hand and arm muscles. Clearly, this refinement should be an essential component of all mirror neuron research, because in one of these monkeys (M43), fully accustomed to the routine of action observation, some sessions did reveal EMG ‘contamination’ during observation sessions, and PTNs recorded during such sessions had had to be excluded from further analysis.

### (d) Could mirror neuron activity be related to orofacial movements?

Other additional controls for movements involving orofacial and ipsilateral hand movements were also carried out (see [12]). F5 neurons with both hand- and mouth-related activity have been reported, sometimes in close proximity, and it is important to check whether the observation-related activity was actually associated with orofacial movements, as we did not record EMG from jaw, tongue or facial muscles. In this study, monkeys were given food rewards 2–3 s after completion of both execution and observation trials, so this was well separated in time from the observed grasping action, and not time-locked to it, because of trial-by-trial variation in delivery of the reward. Therefore, presentation of the reward is unlikely to explain the changes in discharge that occurred consistently around the time the experimenter executed their grasp (figures 1*j*,*k* and 3*h*). Further, we continued to see this discharge even on trials when no food reward was grasped by the experimenter (figure 3*c*) or expected by the monkey. For example, rewards were given on only two of the 10 trials shown in figure 3*g*. The discharge of most F5 and M1 mirror neurons was not modulated by chewing activity and, finally, ICMS delivered at the sites at which we recorded mirror neurons did not evoke orofacial movements. Therefore, we can conclude that it is unlikely that the mirror activity we have recorded was related to the monkey's orofacial movements.

### (e) Mirror neurons in M1

Our results suggest that a significant proportion of PTNs (58%) in primary motor cortex hand area can also show some degree of mirror-like activity. This proportion might seem high compared with earlier reports, but it is important to stress that around a third of our population of M1 mirror neurons (27/77 PTNs) consisted of PTNs whose discharge was suppressed during action observation. In a historical context, it is interesting to note that Gallese *et al*. [16] carried out a different sort of control: they argued that as activity in M1 was known to be movement-related, then the absence of any modulation in discharge in M1 recordings was evidence against the monkey itself making movements while it observed actions. Intriguingly, our findings suggest that there *is* modest mirror-like activity in M1 but it is not associated with overt movement. Further, although the proportion of PTNs responding to action observation can be rather similar in F5 and M1 (figure 3*a–d*), it is clear that in the latter area, responses are quite small and subtle (figure 2*c*,*d*), especially when compared with the very robust changes accompanying the monkey's own grasp. So these responses may have been missed in earlier studies.

### (f) Are the functions of mirror neurons in F5 and M1 the same?

Although all of the neurons we selected for study were PTNs, there are some preliminary lines of evidence to suggest that those in F5 and in M1 could fulfil rather different functions. First, there appear to be clear differences between F5 and M1 PTNs for execution versus observation of a precision grip. Facilitation-type mirror PTNs in F5 showed closely matched firing rates across conditions (figure 2*d*, right, F-F type; cf. [16]), whereas in M1, which is generally considered to be much closer to the motor output, F-F-type neurons were more active for execution than observation (figure 2*d*, left). Second, the mirror neuron population in F5 seems to show a more graded response to grasps carried out in different contexts than does that in M1 (figure 3*a*–*d*). One interpretation might be that more F5 mirror neurons are responsive to the goal of the action, whereas M1 neuron discharge is correlated with the different movements making up the action. A similar conclusion was reached when comparing inferior parietal lobe and F5 neurons [29] and F5 with M1 neurons [18].

These differences in function may well reflect differences in the sub-cortical targets of the PTNs in F5 versus M1. Around 75–80% of M1 PTNs are thought to extend their axons beyond the brainstem to the spinal cord [30,31]. No figure is available for F5 PTNs. F5 corticospinal neurons are far less numerous than in M1 (making up 4% and 50%, respectively, of the total corticospinal output from the frontal lobe [22]). The F5 projection lacks the large, fast-conducting PTNs found in M1 (figure 1*a*). The F5 corticospinal projection is directed mostly to upper cervical segments, with only a weak projection to the cervical enlargement in which the hand muscle motor nuclei are located. The projection from M1 to these motor nuclei is heavy [32,33] and transneuronal retrograde labelling has identified CM cells in the M1 hand area, but not in F5 [34,35]. In this study, we found evidence of post-spike facilitation for some M1 PTNs, but not F5 PTNs.

### (g) Mirror neurons and the withholding of movement

If it is accepted that PTNs in M1 are part of the system that generates active hand movements, then it is important to try to understand how it is that some of these PTNs can be modulated by action observation, but absolutely no movement results, as shown by EMG recording (figure 1*l*).

It is possible that the excitatory inputs from PTNs to spinal interneurons and motoneurons recruited during active grasp could be subjected to presynaptic inhibition and prevented from reaching these targets. This would be difficult to explain for the special case of CM cells, as these inputs are not subjected to presynaptic inhibition [36], suggesting that other systems (e.g. peripheral afferent inputs from the moving limb) do not use this mechanism to modulate or cancel out CM inputs. It is also possible that other descending inputs, inhibitory to hand motoneurons, are more active during observation.

We would speculate that the clue may lie in the activity of M1 PTNs themselves. First, many PTNs are ‘non-mirror’ and, by definition, their outputs are not modulated during observation. Second, some mirror neurons (‘classical’ or facilitation-type PTNs) are only weakly recruited during action observation (figure 2*d*) and, third, suppression mirror neurons are suppressed in this condition. The combined effect of these latter changes is a net disfacilitation of mirror PTN output of over 40 spikes s^−1^ PTN^−1^ (green bar in figure 2*d*). There may be other changes in the temporal structure of mirror PTN output for execution versus observation, reflecting the different ‘neural state’ of the output and its impact on spinal targets [37].

If this view is correct, it suggests that there is a signal that switches PTNs between these two states. It would have to be a fast-switching mechanism, as PTNs can rapidly change their involvement from observation to execution (figure 3*e*). Examples of rapid switching between states have been identified in the oculomotor system [38]. Such a switching mechanism would be very important in activities where two individuals share a skilled task, such as surgery, piano duets and when one person passes an object to another.

## Notes

All experimental procedures were approved by the Local Ethical Procedures committee and carried out in accordance with the UK Animals (Scientific Procedures) Act.

## References

[1] Rizzolatti G Sinigaglia C 2010 The functional role of the parieto-frontal mirror circuit: interpretations and misinterpretations. Nat. Rev. Neurosci. 11, 264–274. (10.1038/nrn2805) 20216547

[2] Jeannerod M Arbib MA Rizzolatti G Sakata H 1995 Grasping objects: the cortical mechanisms of visuomotor transformation. Trends Neurosci. 18, 314–320. (10.1016/0166-2236(95)93921-J) 7571012

[3] Dushanova J Donoghue J 2010 Neurons in primary motor cortex engaged during action observation. Eur. J. Neurosci. 31, 386–398. (10.1111/j.1460-9568.2009.07067.x) 20074212 PMC2862560

[4] Vigneswaran G Philipp R Lemon RN Kraskov A 2013 M1 corticospinal mirror neurons and their role in movement suppression during action observation. Curr. Biol. 23, 236–243. (10.1016/j.cub.2012.12.006) 23290556 PMC3566480

[5] Alegre M Rodriguez-Oroz MC Valencia M Perez-Alcazar M Guridi J Iriarte J Obeso JA Artieda J 2010 Changes in subthalamic activity during movement observation in Parkinson's disease: is the mirror system mirrored in the basal ganglia? Clin. Neurophysiol. 121, 414–425. (10.1016/j.clinph.2009.11.013) 20006544

[6] Fogassi L Ferrari PF Gesierich B Rozzi S Chersi F Rizzolatti G 2005 Parietal lobe: from action organization to intention understanding. Science 308, 662–667. (10.1126/science.1106138) 15860620

[7] Mukamel R Ekstrom AD Kaplan J Iacoboni M Fried I 2010 Single-neuron responses in humans during execution and observation of actions. Curr. Biol. 20, 750–756. (10.1016/j.cub.2010.02.045) 20381353 PMC2904852

[8] Yamazaki Y Yokochi H Tanaka M Okanoya K Iriki A 2010 Potential role of monkey inferior parietal neurons coding action semantic equivalences as precursors of parts of speech. Soc. Neurosci. 5, 105–117. (10.1080/17470910802625306) 20119879 PMC2826156

[9] Rozzi S Ferrari PF Bonini L Rizzolatti G Fogassi L 2008 Functional organization of inferior parietal lobule convexity in the macaque monkey: electrophysiological characterization of motor, sensory and mirror responses and their correlation with cytoarchitectonic areas. Eur. J. Neurosci. 28, 1569–1588. (10.1111/j.1460-9568.2008.06395.x) 18691325

[10] Yoshida K Saito N Iriki A Isoda M 2011 Representation of others’ action by neurons in monkey medial frontal cortex. Curr. Biol. 21, 249–253. (10.1016/j.cub.2011.01.004) 21256015

[11] Vigneswaran G Kraskov A Lemon RN 2011 Large identified pyramidal cells in macaque motor and premotor cortex exhibit thin spikes: implications for cell type classification. J. Neurosci. 31, 14 235–14 242. (10.1523/JNEUROSCI.3142-11.2011) PMC3199219 21976508

[12] Kraskov A Dancause N Quallo MM Shepherd S Lemon RN 2009 Corticospinal neurons in macaque ventral premotor cortex with mirror properties: a potential mechanism for action suppression? Neuron 64, 922–930. (10.1016/j.neuron.2009.12.010) 20064397 PMC2862290

[13] Philipp R Vigneswaran G Lemon R Kraskov A 2012 Macaque gaze behaviour during a grasping task: action execution vs. action observation, Program No. 187.113. 2012, Society for Neuroscience, New Orleans, LA, USA.

[14] Maranesi M Serventi FM Bruni S Bimbi M Fogassi L Bonini L 2013 Monkey gaze behaviour during action observation and its relationship to mirror neuron activity. Eur. J. Neurosci. 38, 3721–3730 (10.1111/ejn.12376) 24118599

[15] Brochier T Spinks RL Umilta MA Lemon RN 2004 Patterns of muscle activity underlying object-specific grasp by the macaque monkey. J. Neurophysiol. 92, 1770–1782. (10.1152/jn.00976.2003) 15163676

[16] Gallese V Fadiga L Fogassi L Rizzolatti G 1996 Action recognition in the premotor cortex. Brain 119, 593–609. (10.1093/brain/119.2.593) 8800951

[17] Quallo MM Kraskov A Lemon RN 2012 The activity of M1 corticospinal neurons during tool use by macaque monkeys. J. Neurosci. 32, 17 235–17 364. (10.1523/JNEUROSCI.1009-12.2012) PMC3678117 23197726

[18] Umilta MA Brochier T Spinks RL Lemon RN 2007 Simultaneous recording of macaque premotor and primary motor cortex neuronal populations reveals different functional contributions to visuomotor grasp. J. Neurophysiol. 98, 488–501. (10.1152/jn.01094.2006) 17329624

[19] Fetz EE Cheney PD 1980 Postspike facilitation of forelimb muscle activity by primate corticomotoneuronal cells. J. Neurophysiol. 44, 751–772. 6253604 10.1152/jn.1980.44.4.751

[20] Lemon RN Mantel GW Muir RB 1986 Corticospinal facilitation of hand muscles during voluntary movement in the conscious monkey. J. Physiol. 381, 497–527. 3625543 10.1113/jphysiol.1986.sp016341 PMC1182993

[21] di Pellegrino G Fadiga L Fogassi L Gallese V Rizzolatti G 1992 Understanding motor events: a neurophysiological study. Exp. Brain Res. 91, 176–180. 1301372 10.1007/BF00230027

[22] Dum RP Strick PL 1991 The origin of corticospinal projections from the premotor areas in the frontal lobe. J. Neurosci. 11, 667–689. 1705965 10.1523/JNEUROSCI.11-03-00667.1991 PMC6575356

[23] Borra E Belmalih A Gerbella M Rozzi S Luppino G 2010 Projections of the hand field of the macaque ventral premotor area F5 to the brainstem and spinal cord. J. Comp. Neurol. 518, 2570–2591. (10.1002/cne.22353) 20503428

[24] He SQ Dum RP Strick PL 1993 Topographic organization of corticospinal projections from the frontal lobe: motor areas on the lateral surface of the hemisphere. J. Neurosci. 13, 952–980. 7680069 10.1523/JNEUROSCI.13-03-00952.1993 PMC6576595

[25] Belmalih A Borra E Contini M Gerbella M Rozzi S Luppino G 2009 Multimodal architectonic subdivision of the rostral part (area F5) of the macaque ventral premotor cortex. J. Comp. Neurol. 512, 183–217. (10.1002/cne.21892) 19003790

[26] Caggiano V Fogassi L Rizzolatti G Thier P Casile A 2009 Mirror neurons differentially encode the peripersonal and extrapersonal space of monkeys. Science 324, 403–406. (10.1126/science.1166818) 19372433

[27] Caggiano V Fogassi L Rizzolatti G Pomper JK Thier P Giese MA Casile A 2011 View-based encoding of actions in mirror neurons of area f5 in macaque premotor cortex. Curr. Biol. 21, 144–148. (10.1016/j.cub.2010.12.022) 21236674

[28] Caggiano V Fogassi L Rizzolatti G Casile A Giese MA Thier P 2012 Mirror neurons encode the subjective value of an observed action. Proc. Natl Acad. Sci. USA 109, 11 848–11 853. (10.1073/pnas.1205553109) PMC3406819 22753471

[29] Bonini L Rozzi S Serventi FU Simone L Ferrari PF Fogassi L 2010 Ventral premotor and inferior parietal cortices make distinct contribution to action organization and intention understanding. Cereb. Cortex 20, 1372–1385. (10.1093/cercor/bhp200) 19805419

[30] Humphrey DR Corrie WS 1978 Properties of pyramidal tract neuron system within a functionally defined subregion of primate motor cortex. J. Neurophysiol. 41, 216–243. 413887 10.1152/jn.1978.41.1.216

[31] Porter R Lemon RN 1993 Corticospinal function and voluntary movement. Oxford, UK: Oxford University Press.

[32] Morecraft RJ Ge J Stilwell-Morecraft KS McNeal DW Pizzimenti MA Darling WG 2013 Terminal distribution of the corticospinal projection from the hand/arm region of the primary motor cortex to the cervical enlargement in rhesus monkey. J. Comp. Neurol. 521, 4205–4235. (10.1002/cne.23410) 23840034 PMC3894926

[33] Armand J Olivier E Edgley SA Lemon RN 1997 Postnatal development of corticospinal projections from motor cortex to the cervical enlargement in the macaque monkey. J. Neurosci. 17, 251–266. 8987753 10.1523/JNEUROSCI.17-01-00251.1997 PMC6793701

[34] Rathelot JA Strick PL 2009 Subdivisions of primary motor cortex based on cortico-motoneuronal cells. Proc. Natl Acad. Sci. USA 106, 918–923. (10.1073/pnas.0808362106) 19139417 PMC2621250

[35] Rathelot JA Strick PL 2006 Muscle representation in the macaque motor cortex: an anatomical perspective. Proc. Natl Acad. Sci. USA 103, 8257–8262. (10.1073/pnas.0602933103) 16702556 PMC1461407

[36] Jackson A Baker SN Fetz EE 2006 Tests for presynaptic modulation of corticospinal terminals from peripheral afferents and pyramidal tract in the macaque. J. Physiol. 573, 107–120. (10.1113/jphysiol.2005.100537) 16556658 PMC1779692

[37] Shenoy KV Sahani M Churchland MM 2013 Cortical control of arm movements: a dynamical systems perspective. Annu. Rev. Neurosci. 36, 337–359. (10.1146/annurev-neuro-062111-150509) 23725001

[38] Isoda M Hikosaka O 2008 Role for subthalamic nucleus neurons in switching from automatic to controlled eye movement. J. Neurosci. 28, 7209–7218. (10.1523/JNEUROSCI.0487-08.2008) 18614691 PMC2667154

